# Dr. Ward Cousins on the Century's Progress

**Published:** 1899-08-05

**Authors:** 


### Dr. Ward Cousins on the Century's Progress.

In the present state of the weather, in the midst of the Cowes week, and with the beautiful Solent rippling in the welcome breeze, it certainly must have been a trying task to have to deliver an address such as custom now demands from the President of the British Medical Association on the occasion of its annual meeting. Nor was the task made easier by the restrictions which custom has imposed upon it. Each "Section" opens with its own address, its own little *résumé* of the year's work in its own department, and, in addition, there are set addresses on medicine and on surgery; and it has come to be expected that the president in framing his remarks will not trench too much upon the reserves of these various departments, and will not knock the bottom out of the addresses which are to follow. Hence perhaps it is that Dr. Ward Cousins, who could have dealt so well with many a surgical subject on which he has worked himself, has felt bound to offer to his guests a salmagundi, a medley of mingled topics, on all of which he speaks pleasantly, and on few with any too much authority. The result, it must be confessed, is disappointing. The address is full of information, historical and otherwise—which, again, like history, is veracious and otherwise—but there is a sad absence of pungency and pepper. "Kindly go back with me," says the president, "to dawn of the present century, and take a hasty survey of the general condition of medical science at that time." So back we go, and we take our survey, and are introduced again to John Hunter, his "brilliant researches," his "extraordinary industry," and his "patient study," all of which is good, but we think we have heard it before. Then, lest Scotland should be "damned with faint praise," we are told about Gregory and Cullen, the latter of whom by his wisdom "captivated the medical mind of Europe." With that admirable impartiality which is necessary in the president of so cosmopolitan a gathering, we are also told that "Ireland, too, helped in the progress of our science," and the names of Graves and Stokes are given as instances of her genius. Then Edward Jenner and vaccination have their little turn,; and, after that, we come back to the progress of sanitary science and preventive medicine. Again we find ourselves at the beginning of the century, and working onwards from the "narrow courts, the stifling slums, the unwholesome factories"—which we are left to imagine no longer exist—with the accompanying "disease, deformity, moral and physical degeneration," and other evils caused by these conditions, we are led by way of the honoured names of Farr, and Parkes, and Florence Nightingale, to Ernest Hart and the *British Medical Journal*. From this climax we at once drop back to another subject, and the history of pathology is then traced out, again starting with the inevitable John Hunter. The same thing is done once more for bacteriology, all the well-known names being set forth afresh, and so on. The discovery of surgical anæsthesia and its influence on surgical practice forms quite a chapter in this discourse, and to those who had not got it all at their finger ends already, this part of the address must have been most interesting. Nor should the same praise be denied to the portion devoted to what we may call the current novelties of the day, the mosquitos, the malaria parasite, the relation between the disorders of mankind and those of the lower animals, &c. All these were set forth in a most complete and interesting manner, and all with that same even-handed impartiality which equally favours all men. Such as it was, however, the address was a great success, and well suited the occasion on which it was delivered. There is a home-like feeling in knowing pretty well what sort of an address one is going to hear; and so we like these annual meetings of the British Medical Association, for all their heat and squash, and for all their refurbishing of ancient scientific armour. If space allowed, we could quote many passages from Dr. Ward Cousins's address which would show how thoroughly he had entered into the wide researches necessary for its production, and many others which would show how well he demonstrated and made clear the wider bearings of the facts with which he dealt; but to tell the truth, good as he was as a showman, and nimbly as he made his little figures trot across the stage while he described the progress of the century, we had rather that Ward Cousins himself had been the subject of the address, and that he had related his own researches, his own work, his own doings in connection with his hospital, and especially that he had described his own inventions and the lines of thought which set him to devise all the useful contrivances associated with his name, which have done so much to comfort his patients and to facilitate the surgeon's work. A man who invents is always a man who has found difficulties to overcome, and we venture to think that he would have held his audience more enthralled, even than he did by the historical summary which he gave, had he told them more of the surgical difficulties which he had met with, and of the labour which he had bestowed in lessening these difficulties for those who would follow him. The personal is always interesting. The personal, however, with praiseworthy but regrettable modesty, was suppressed.

